# Epidemiology of rheumatoid arthritis in sub-Saharan Africa: a systematic review and meta-analysis protocol

**DOI:** 10.1186/s13643-020-01342-5

**Published:** 2020-04-17

**Authors:** Mickael Essouma, Jan René Nkeck, Francky Teddy Endomba, Jean Joel Bigna, Stéphane Ralandison

**Affiliations:** 1 Department of Internal Medicine and Specialties, Faculty of Medicine and Biomedical Sciences, University of Yaoundé I, Yaoundé, Cameroon; 2 Psychiatry internship program, University of Bourgogne, 21000 Dijon, France; 3 Department of Epidemiology and Public Health, Centre Pasteur of Cameroon, Yaoundé, Cameroon; 4 School of Public Health, Faculty of Medicine, University of Paris Sud XI, Le Kremlin Bicêtre, France; 5 Department of Internal Medicine and Division of Rheumatology, Toamasina University Teaching Hospital and Faculty of Medicine of Toamasina, Tamatave, Madagascar

**Keywords:** Rheumatoid arthritis, Epidemiology, Sub-Saharan Africa

## Abstract

**Background:**

So far, only one meta-analysis has estimated the prevalence of rheumatoid arthritis (RA) in Africa. Out of 10 studies included in that meta-analysis, nine came from sub-Saharan African countries and had been published between 1968 and 1988. We will conduct a new systematic review and meta-analysis to update their estimates and provide more consistent prevalence data on RA in sub-Saharan Africa.

**Methods:**

We will comprehensively search electronic databases to select observational studies addressing RA in sub-Saharan Africa and published as from 1 January 2000: PubMed, EMBASE, African Journals Online, Web of Science, and Global Index Medicus. Summary estimates will be derived through random-effects meta-analysis whenever possible. Alternatively, estimates will be reported through narrative synthesis when the random-effects meta-analysis will be impossible. The risk of bias will be assessed using standard methods.

**Discussion:**

This systematic review and meta-analysis shall quantify the magnitude of RA morbidity and mortality in sub-Saharan Africa. Results from this review will be disseminated in peer-reviewed journals, conferences and on social media platforms.

**Systematic review registration:**

PROSPERO CRD42020153483

## Background

Rheumatoid arthritis (RA) is the prototypical chronic inflammatory rheumatic disease, affecting 5 to 10 per 1000 adults in industrialized countries. Delayed care leads to severe RA, with ensuing physical disability, poor quality of life and premature death [1]. Of note, RA was responsible for 3.4 million disability-adjusted life years globally during 1990-2017 [2]. Furthermore, up to 50% more deaths are recorded in RA patients than in the general population [3–6]. To mitigate RA impact to the population, early detection and treatment of prevalent cases are paramount. 

To the best of our knowledge, there are no real-life contemporary estimates of the prevalence of RA in sub-Saharan Africa. Indeed, the initial pooled prevalence of 0.36% for Africa [7] was based on data from nine (out of 10 included) studies originating from sub-Saharan Africa and published during 1968-1988. Recently, age-standardized prevalence rates of 135.7 to 231.1 per 100 000 sub-Saharan African population were generated from statistical modelling for the Global Burden of Disease study [2]. In light of these data [2, 7], the apparent rarity of RA [2, 7–9] may be biased towards substantial under-investigation and underdiagnosis of this disease within sub-Saharan Africa during the last century. Moreover, RA prevalence is likely increasing within the region, as a growing body of evidence supports an epidemiological transition with rise in the burden of autoimmune rheumatic diseases in sub-Saharan Africa [10–12].

Taken together, there is an urgent need for contextual updated prevalence data to inform policies aimed at early identification and management of RA in sub-Saharan Africa. Rudan et al [9] failed to provide such data in their meta-analysis due to the lack of new relevant knowledge as from the time Dowman et al’s article [7] was released, to the end of their search dates [9]. Accordingly, we will conduct a systematic review and meta-analysis aiming to update previous pooled estimates and provide more consistent prevalence data on RA in sub-Saharan Africa.

## Methods and design

### Design

We will follow the PRISMA (Preferred Reporting Items for Systematic Review and Meta-analyses) guidelines for systematic reviews and meta-analyses [13]. This protocol follows the PRISMA-P (Preferred Reporting Items for Systematic Review and Meta-analysis Protocols) guidelines [14].

### Eligibility criteria

#### Inclusion criteria

1. Types of studies: published observational studies (case series, cross-sectional, case-control, and cohort studies) reporting on RA. The classification for RA should be based on the 1987 American College of Rheumatology (ACR) and/or 2010 ACR/European League Against Rheumatism criteria [15, 16].
2. Participants: sub-Saharan Africans living in sub-Saharan Africa according to the geographic classification of the United Nations Statistics Division [17], regardless of gender and age.
3. Outcome: prevalence of RA, RA comorbidities, and mortality.

#### Exclusion criteria

1. Studies not performed in humans.
2. Studies published in languages other than English or French.
3. Studies with sample size less than 10.
4. Case reports, commentaries, reviews, editorials, and studies without primary data or explicit description of methods, or both.
5. Qualitative studies.
6. Studies that lack relevant data needed to compute the prevalence of RA, RA comorbidities, and mortality.
7. Studies on mixed connective tissue disease or other overlapping syndromes.

### Information sources and search strategy

A comprehensive search of electronic databases will be done to select observational studies reporting on the prevalence of RA, RA comorbidities, and mortality in sub-Saharan Africa and published as from 1 January 2000: PubMed, EMBASE, African Journals Online, Web of Science, and Global Index Medicus. There will be no language restriction. We will restrict the search to the last 20 years to have updated and robust data. The main strategy that will be applied in PubMed is shown in Additional file 1: Table S1. This will be adapted for the search in other databases. The electronic search will be followed by hand searches to identify any studies missed during the review process or by the search strategy.

### Study selection

Duplicates will be removed using EndNote 7. Two investigators will independently assess the titles and/or abstracts of the retrieved papers for eligibility using the Rayyan online application. Two investigators will independently evaluate the full texts of the selected records. Discrepancies will be resolved by consensus or will involve a third author as an arbitrator. The agreement between the two investigators will be estimated by Cohen’s kappa coefficient [18]. For duplicates, the study with the largest sample size will be considered. Studies whose full texts will not be available even after the author request will not be considered.

### Assessment of the risk of bias in individual studies

Two investigators will independently appraise the quality of individual studies using the 10-item rating system (Additional file 1: Table S2) proposed by Hoy et al. [19]. Each item will be assigned a score of 1 (yes) or 0 (no). The total score for each study will be generated by summing individual items’ scores, in order to obtain a quality score ranging from 0 to 10. A study will be considered at low risk of bias if its quality score is higher than 8, at moderate risk if its score ranges from 6 to 8, and at high risk if its score is equal to or lower than 5. Disagreements between the two investigators shall be resolved through consensus or with a third investigator as arbitrator.

### Data extraction and management

Two investigators will independently extract data to minimize reporting or classification errors, and disagreements will be solved with a third investigator as an arbitrator or by consensus. Data to be extracted are the name of the first author, year of publication, study design, period of recruitment of the study population, country, locality (urban/rural), setting (hospital-based/community-based), sampling method, total number of participants, classification criteria for RA, number of participants with RA, and outcome of interest aforesaid.

### Data synthesis and analysis

For the derivation of summary prevalence estimates, a random-effects model will be used whenever possible, according to Barendregt et al.’s method [20]. To minimize the influence of studies with extremely large prevalence estimates on the overall estimate, the variance of study-specific prevalence will be stabilized with the Freeman-Turkey double arcsine transformation before pooling through the random-effects model [20]. We will additionally perform sensitivity analyses including only studies with sample size above 50 to assess the role of the sample size on the precision of the prevalence estimate. A narrative synthesis will be the alternative option to report prevalence estimates when the meta-analysis will be impossible. These meta-analyses will be conducted with the “*meta*” packages of the R statistical software (version 3.5.1, the R statistical Foundation for statistical computing, Vienna, Austria).

### Assessment of the risk of bias across studies

The Cochran’s *Q* statistics will be used to assess inter-study heterogeneity [21], and *I*^*2*^ will be used to quantify the degree of inconsistency by calculating the percentage of total variation due to heterogeneity rather than chance. *I*^*2*^ values of < 25%, 25–75%, and > 75% will correspond to low, moderate, and high heterogeneity, respectively. The Cochran’s *Q* statistics will be two-tailed, with *p* < 0.05 considered statistically significant. The risk of publication bias will be evaluated using the Egger’s linear regression, with *p* < 0.1 defining publication bias [22].

### Potential amendments

We do not intend to modify this protocol. However, any potential modification shall be reported in the final publication.

### Ethics and dissemination

This work does not require an ethical approval since it is based on published data. Results from this systematic review and meta-analysis will be shared in peer-reviewed journals, conferences and on social media platforms. We also plan future update of our results.

## Discussion

This systematic review and meta-analysis is destined to capture the contemporary magnitude of RA morbidity and mortality in sub-Saharan Africa for informing RA diagnosis and management strategies within this region. This systematic review and meta-analysis will also serve as an accurate basis for future relevant research in sub-Saharan Africa.

This work may have limitations that are commonly encountered in systematic reviews on autoimmune rheumatic diseases in the region [10, 23]: country underrepresentativeness, high inter-study heterogeneity as well as limited number of large, community-based and longitudinal studies. Needless to say, a selection bias is likely, especially as this upcoming systematic review will only be based on articles published in English and French languages [24].

## Supplementary information

**Additional file 1: Table S1.** Search strategy for PubMed. **Table S2.** Criteria for quality assessment in prevalence studies.

## Data Availability

Further information related to this review protocol can be provided upon reasonable request. Interested readers should contact the corresponding author.

## Abbreviations

ACR: American College of Rheumatology
PRISMA: Preferred Reporting Items for Systematic Review and Meta-analyses
PRISMA-P: Preferred Reporting Items for Systematic Reviews and Meta-analysis Protocols
RA: Rheumatoid arthritis
