# Supplementary material for: Epidemiology of rheumatoid arthritis in sub-Saharan Africa: a systematic review and meta-analysis protocol
Source: Syst Rev. 2020 Apr 17;9:81. doi: 10.1186/s13643-020-01342-5 (PMC7164226; doi:10.1186/s13643-020-01342-5)
Supplement: Supplementary file 1 — Additional file 1: Table S1. Search strategy for PubMed. Table S2. Criteria for quality assessment in prevalence studies. [file 13643_2020_1342_MOESM1_ESM.docx]

**Appendix**

[Table 1: Search strategy for PubMed 1](#_Toc35691850)

[Table 2: Criteria for quality assessment in prevalence studies 2](#_Toc35691851)

[Reference 3](#_Toc35691852)

# **Table 1: Search strategy for PubMed**

| **Search** | **Search terms** |
| --- | --- |
| #1 | “rheumatoid arthritis” OR “rheumatoid polyarthritis” OR “juvenile rheumatoid arthritis” OR “juvenile rheumatoid polyarthritis” OR “felty syndrome” or “rheumatoid vasculitis” OR “Sjogren’s syndrome” OR “still’s disease” OR “rheumatoid nodule” |
| #2 | Africa* OR Angola OR Benin OR Botswana OR "Burkina Faso" OR Burundi OR "Cabo Verde" OR Cameroon OR "Canary Islands" OR "Central African Republic" OR Chad OR Comoros OR Congo OR "Democratic Republic of the Congo" OR Djibouti OR "Equatorial Guinea" OR Eritrea OR Eswatini OR Ethiopia OR Gabon OR Gambia OR Ghana OR Guinea OR "Guinea Bissau" OR "Ivory Coast" OR "Cote d’Ivoire" OR Kenya OR Lesotho OR Liberia OR Madagascar OR Malawi OR Mali OR Mauritania OR Mauritius OR Mayotte OR Mozambique OR Namibia OR Niger OR Nigeria OR Réunion OR Rwanda OR "Sao Tome and Principe" OR Senegal OR Seychelles OR "Sierra Leone" OR Somalia OR "South Africa" OR “South Sudan” OR "Saint Helena" OR Togo OR Uganda OR “United Republic of Tanzania” OR Zambia OR Zimbabwe OR "Eastern Africa" OR “Middle Africa” OR "Southern Africa" OR "Western Africa" OR "East Africa" OR "East African" OR "Eastern Africa" OR "South African" OR "Southern African" OR "West Africa" OR "West African" OR "Western African" OR "Central Africa" OR "Central African" OR "Western Sahara" OR "sub Saharan Africa" OR "sub Saharan African" OR "subSaharan Africa" OR "subSaharan African" |
| #3 | #1 AND #2 |
| #4 | #Limit [2000-[ |

# **Table 2: Criteria for quality assessment in prevalence studies**

| **Items** | **Quality score** |
| --- | --- |
| **External validity** |  |
| 1. Was the study’s target population a close representation of the national population in relation to relevant variables? | (1 point) |
| 2. Was the sampling frame a true or close representation of the target population? | (1 point) |
| 3. Was some form of random selection used to select the sample, OR was a census undertaken? | (1 point) |
| 4. Was the likelihood of nonresponse bias minimal? | (1 point) |
|  | Total (4 points) |
| **Internal validity** |  |
| 5. Were data collected directly from the subjects (as opposed to a proxy)? | (1 point) |
| 6. Was an acceptable case definition used in the study? | (1 point) |
| 7. Was the study instrument that measured the parameter of interest shown to have validity and reliability? | (1 point) |
| 8. Was the same mode of data collection used for all subjects? | (1 point) |
| 9. Was the length of the shortest prevalence period for the parameter of interest appropriate? | (1 point) |
| 10. Were the numerator(s) and denominator(s) for the parameter of interest appropriate? | (1 point) |
|  | Total (6 points) |

Source: Hoy et al **[1]**

# **Reference**

1. Hoy D, Brooks P, Woolf A, Blyth F, March L, Bain C, et al. Assessing risk of bias in prevalence studies: modification of an existing tool and evidence of interrater agreement. J Clin Epidemiol. 2012;65(9):934‑9.
